# Noncontact Respiratory Monitoring Using Depth Sensing Cameras: A Review of Current Literature

**DOI:** 10.3390/s21041135

**Published:** 2021-02-06

**Authors:** Anthony P. Addison, Paul S. Addison, Philip Smit, Dominique Jacquel, Ulf R. Borg

**Affiliations:** 1 Medtronic, Video Biosignals Group, Patient Monitoring, Edinburgh EH26 0PJ, UK; 2311417A@student.gla.ac.uk (A.P.A.); philip.smit@medtronic.com (P.S.); dominique.jacquel@medtronic.com (D.J.); 2 Medtronic, Medical Affairs, Patient Monitoring, Boulder, CO 80301, USA; ulf.borg@medtronic.com

**Keywords:** noncontact monitoring, depth-sensing camera, respiratory monitoring, respiratory rate, tidal volume, respiratory patterns, pandemic monitoring

## Abstract

There is considerable interest in the noncontact monitoring of patients as it allows for reduced restriction of patients, the avoidance of single-use consumables and less patient–clinician contact and hence the reduction of the spread of disease. A technology that has come to the fore for noncontact respiratory monitoring is that based on depth sensing camera systems. This has great potential for the monitoring of a range of respiratory information including the provision of a respiratory waveform, the calculation of respiratory rate and tidal volume (and hence minute volume). Respiratory patterns and apneas can also be observed in the signal. Here we review the ability of this method to provide accurate and clinically useful respiratory information.

## 1. Introduction

The measurement of respiratory physiological parameters is ubiquitous in the hospital setting. Of these, respiratory rate (RR) is the most often measured and recorded, and forms an essential component of many early warning clinical scoring systems such as MEWS, NEWS, etc. [1]. Changes in RR are often one of the earliest and more important indicators that precedes major complications such as respiratory tract infections, respiratory depression associated with opioid consumption, anaesthesia and/or sedation, as well as respiratory failure [2,3,4]. A wide range of methods have been proposed for the determination of respiratory rate using noncontact means including RGB video camera systems [5,6], infrared camera systems [7], laser vibrometry [8], piezoelectric bed sensors [9], Doppler radar [10], thermal imaging [11], acoustic sensors [12], and radio frequency methods (radar and WiFi) [13]. Tidal volume (TV) is less often measured at the bedside, as it requires a measurement of airflow from the patient’s mouth which necessitates a sealed mask or intubation. However, along with its counterparts of RR, SpO2 and PaCO_2_, it is recognised as a critical parameter in understanding pathophysiologic patterns of death which evolve due to sepsis, congestive heart failure, pulmonary embolism, hypoventilation, narcotic overdose, and sleep apnea [14].

Depth cameras have proved adept at capturing the motion associated with respiration. From the resulting respiratory volume (RV) signal, measures of both respiratory rate and tidal volume can be made. This has prompted an interest in the use of such cameras for providing these physiological parameters. Moreover, the combination of RR and TV, combined within a noncontact modality, could provide a valuable monitoring tool during viral pandemics, including novel coronavirus (COVID-19) patients as well as those with other viral respiratory tract diseases where minimum contact with the patient is desired [15]. It has been emphasised by Massarroni et al. [16] that respiratory rate can be poorly recorded despite its relevance in the context of COVID-19.

This review of the literature considers the implementation of depth cameras in the noncontact physiological monitoring of respiratory information. The technology has great potential for the noncontact monitoring of a range of respiratory and contextual patient parameters, including respiratory rate, tidal volume and minute volume trending, respiratory pattern identification, apnea detection, motion activity, presence in bed, etc. In addition, noncontact monitoring allows for reduced restriction of patients, the avoidance of single-use consumables and the reduction of the spread of disease. Here, the performance of depth-sensing systems in the measurement of respiratory parameters is reviewed in detail.

This review was conducted by manual inspection of a Google Scholar search including various combinations of the terms: ‘respiratory’; ‘tidal volume’; ‘depth sensing’; ‘camera’; ‘monitoring’, etc. These were reviewed manually and those that corresponded to the aim of the literature review were selected for inclusion.

## 2. Deriving Respiratory Information Using a Depth Camera

**Figure 1:**
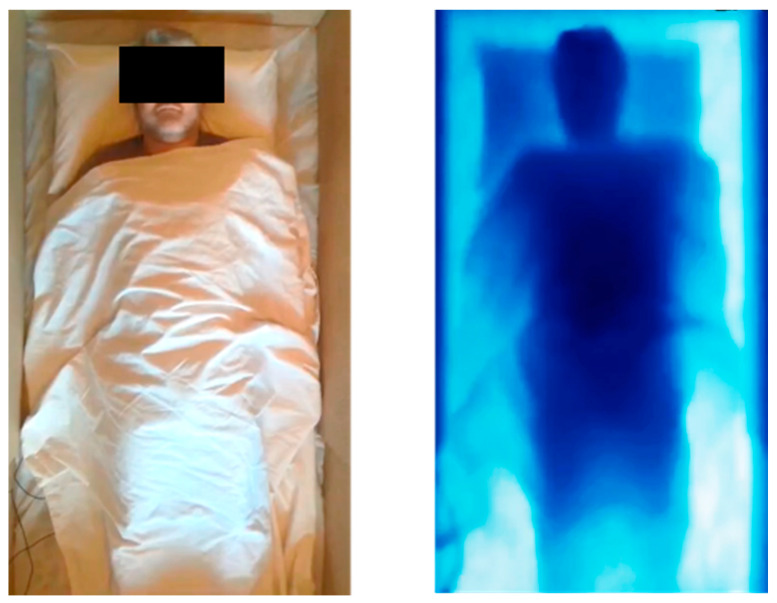
An RGB image of a subject under a sheet (**left**) and its corresponding depth image (**right**).

A depth camera measures the distance to the surface of all objects within its field of view (FOV) and outputs a single matrix of distances (or depths) for each image frame. This is in contrast to an RGB image, used for photographs and video feeds, which comprises three matrices encoding colours in the scene: red, green and blue (RGB). As an example of the difference between these two modalities, Figure 1 shows the RGB and depth images taken of the same scene of a subject lying supine under covers. To view the final depth information, we are required to convert the depth values to a false colour image. Here, a blue scale was used with the darker blue representing surfaces nearer to the camera.

Depth camera systems may be based on one of stereoscopic, structured light or time-of-flight principles. Stereoscopic depth cameras resolve depth through two slightly different perspective views of the same scene. This method is similar to the manner in which frontal vision animals perceive depth. Algorithmically, depth is constructed from the two views by calculating the disparities between features or key points in the scene. Depth cameras operating on the time-of-flight principle, measure distance to points in the scene by measuring the time it takes for a signal emitted from the camera to return due to reflection. The scene is thus actively illuminated by the camera’s emitter (usually an IR laser) and recovers the distance information either through a direct (i.e., half the return time) or indirect (i.e., phase recovery of a modulated emitter signal) method. Structured light and the related coded light-based cameras project a (usually IR) pattern onto a scene. The pattern, a series of stripes for example, is known and depth is obtained by analysing the deformation caused by the scene and perceived by the camera.

**Figure 2:**
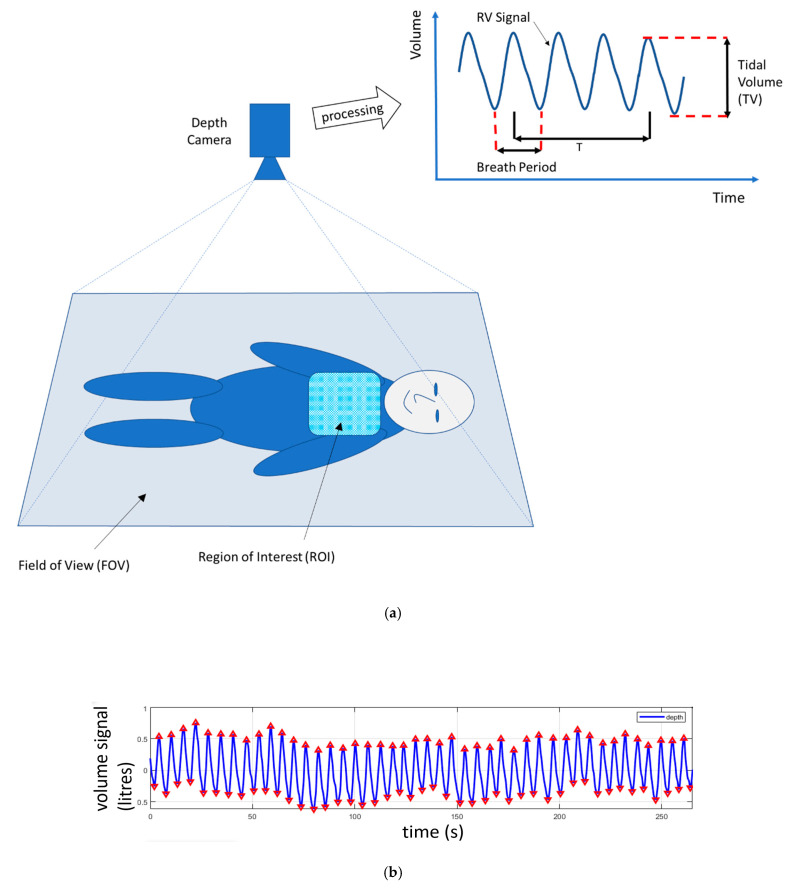
(**a**) Depth camera system schematic illustrating the field of view (FOV) and region of interest (ROI) and the processing of the ROI information to produce the respiratory volume (RV) signal. T is the length of a time window used in the calculation of respiratory rate. (**b**) Respiratory volume signal acquired from a heathy volunteer using a Kinect^TM^ V2 depth camera (Microsoft, Redmond, WA, USA). Triangles indicate the peaks and troughs of the signal modulations.

**Figure 3:**
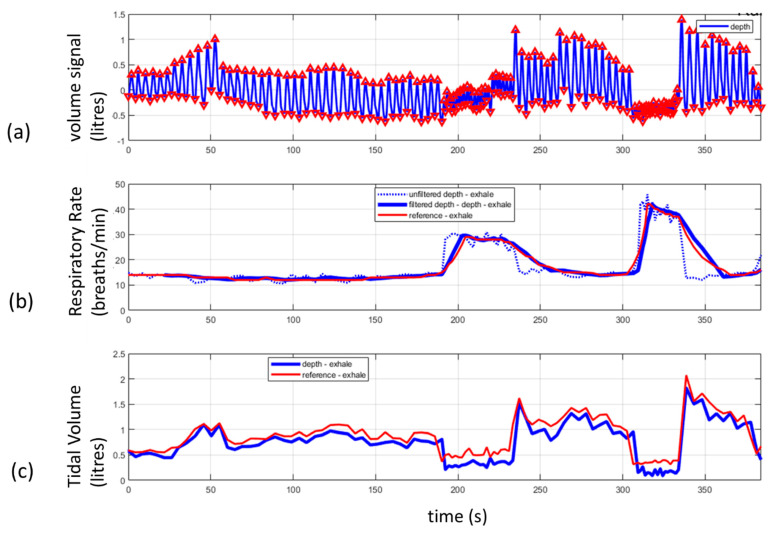
An example of respiratory rate and tidal volume derived from a depth sensing system. (**a**) The volume signal obtained from a depth sensing camera. The signal was generated by the subject varying his tidal volume over time. (**b**) The raw and filtered respiratory rate from a depth system compared to a ventilator reference. (**c**) The tidal volume computed from the respiratory volume signal (peak to trough in (**a**)) compared to a ventilator reference.

When directed at a patient’s chest region, the changes in distances to the chest as the patient breathes can be used to calculate a respiratory volume signal (RV signal). This is achieved by integrating the depth changes across a ROI defined on the patient’s chest. This summing up of the distances across the area of the region of interest produces a volume change between each frame of the depth image. This volume change may then be used to produce a volume signal over time: the RV signal. A schematic of an RV signal derived in this way is shown in Figure 2a. The ROI may be selected by the camera user by defining a simple rectangular region on the scene which includes some, or all, of the chest region. In some cases, the whole scene in the field of view may be used as the ROI and in other cases a bespoke, nonrectangular ROI which better fits the area of interest may be used. (Examples of various ROIs are provided in the next paragraph.) A number of physiological parameters can be determined from this respiratory volume signal including the tidal volume and respiratory rate. An estimate of TV may be determined from the difference in volume between the peak and the trough of the RV signal, and the RR is calculated from the number of RV signal modulations (breaths) occurring within a window of period T. These measurements are depicted schematically in Figure 2a. An example of a respiratory volume signal from a healthy volunteer is shown in Figure 2b. This was acquired from a healthy adult subject lying supine and breathing at a rate of around 10–12 breaths per minute. Figure 3 contains an example of respiratory rate and tidal volume measurements calculated from the respiratory volume signal taken from a healthy volunteer using a Kinect depth camera. Figure 3a shows the respiratory volume signal obtained as the volunteer undertook various respiratory volume changes over time. The peaks and troughs of the individual breaths are marked by triangles in the plot. Figure 3b shows the instantaneous respiratory rate (per breath) and its equivalent smoothed rate to mimic the smoothing of the reference signal taken from a ventilator. In this way we could match the filtering characteristics of the respiratory rate reported by the ventilator. Figure 3c shows the tidal volume calculated from the respiratory volume signal compared to the tidal volume measured by the ventilator.

**Figure 4:**
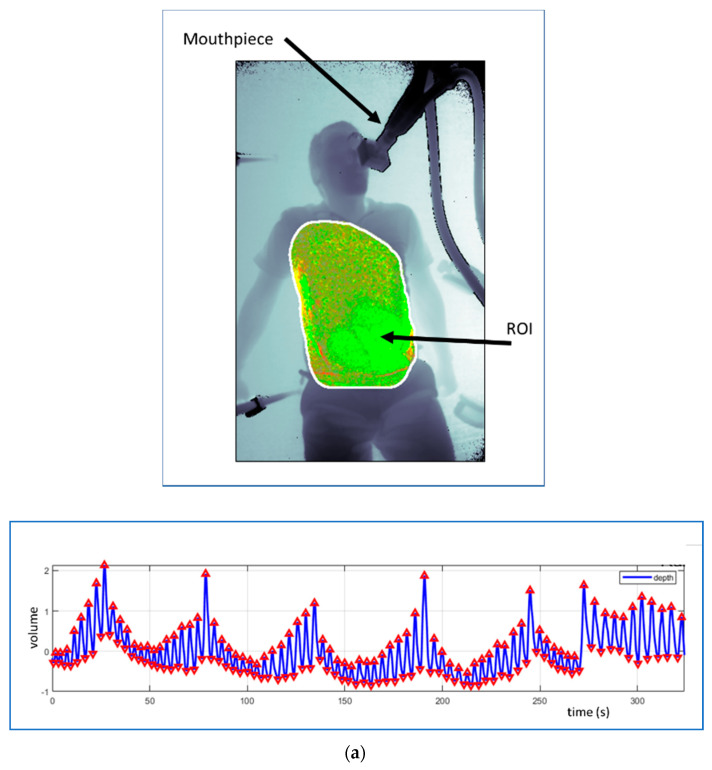

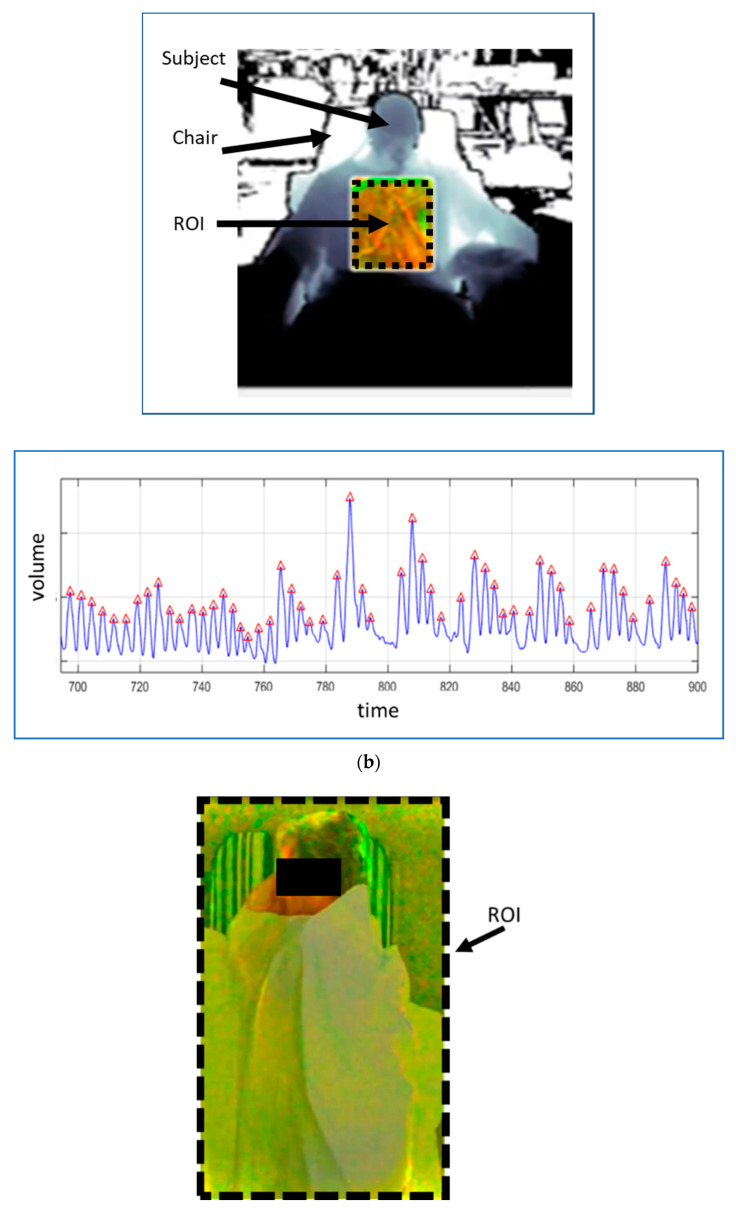

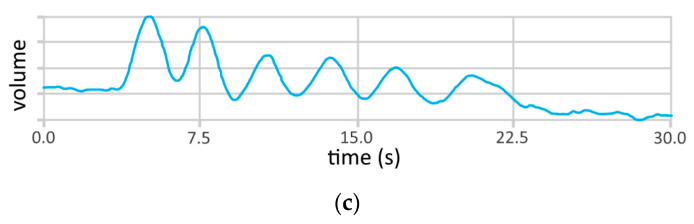
Examples of respiratory patterns in the RV signal from three separate studies. (**a**) Respiratory patterns generated by deliberately varying tidal volume cyclically. The ROI is fitted to the chest region using a flood fill technique. (**b**) Respiratory patterns manifest in a signal collected during a breathe-down study. The ROI is a rectangular subset of the chest. (Reprinted from [17].) (**c**) A simulated apnea signal. The ROI is the whole image.

Respiratory patterns can be observed in the RV signal, including reductions in tidal volume and apneic events. Figure 4 contains some illustrative examples of respiratory patterns evident in the respiratory volume signal together with the various ROIs used to generate them. Figure 4a shows the respiratory pattern generated during the same experimental set up as Figure 2a. A healthy subject lay supine on the floor and breathed at varying tidal volumes over a cyclical pattern. The figure also contains the depth image of the subject with the ROI used to capture the data indicated. We can see that the ROI in this case was fitted to the chest and abdominal region. Figure 4b shows the respiratory pattern obtained during a breathe-down study conducted as part of a pulse oximetry trial [17]. In this study, volunteers were fitted with a face mask in order to adjust their FiO_2_ levels using a mixture of nitrogen and oxygen to induce desaturation. The Cheynes–Stokes-like respiratory pattern shown in Figure 4b was exhibited by one of the subjects during an induced hypoxic episode within the study. In this case, the ROI was confined to a small rectangular region within the chest of the subject. Figure 4c contains one of a sequence of cyclical apnea events generated by a healthy volunteer. This was done to simulate the repetitive reductions in airflow signals observed in sleep apnea patients. The periodic absence of respiratory activity is apparent in the respiratory volume signal shown. Note that the whole field of view was used as the ROI in order to generate this RV signal. It should be noted that the quality, and quantity (!), of the RV signal depends on the choice of ROI. A wide ROI may take in background motions and cause noise on the RV signal. A smaller ROI, e.g., a subchest region, may be relatively noise free but not adequately represent the full tidal volume. An optimal method may be to detect the whole chest region through, for example, a flood fill or other automated method. This may appeal in theory, but these methods must accurately track the ROI and if not, can cause their own issues resulting in the degradation of RV signal quality. Clothing and/or bed sheets and blankets may also cause the derived tidal volumes to not match actual tidal volumes. However, the signals derived will scale with tidal volume and thus provide accurate respiratory rate determination and the display of respiratory patterns. In addition, movement of the subject can cause large scale volume changes and should be dealt with in the post-processing phase of physiological parameter determination. Motion artefact is, of course, something that most physiological monitoring, contact and noncontact, must deal with [18].

## 3. Respiratory Rate

Depth-sensing respiratory rate studies fall into two reasonably distinct categories: (1) benchtop or laboratory studies and (2) clinical healthy volunteer and patient studies. These are discussed in turn as follow:

### 3.1. Respiratory Rate Benchtop/Lab Studies

These studies usually comprise ad hoc investigations conducted with the purpose of understanding key fundamental aspects of the technology or providing limited proof of principle. They usually involve a limited number of volunteers (often just a single volunteer) and limited recording and/or analysis.

An early study by Benetazzo et al. [19] involved five healthy volunteers who took part in various experiments. These included preliminary tests to choose the best sampling frequency, validation tests and finally robustness tests to evaluate the performance of the algorithm in different operating conditions (i.e., orientation of the person, lighting condition and type of clothing). All operating conditions produced high Pearson correlations (with a minimum correlation of 0.93 obtained at low lighting conditions) between breaths recorded over a minute using a spirometer and their depth system; thus, it was concluded that such a system could be used for measuring human respiratory rate. Another study by Dang et al. [20] utilised a Prime Sense camera (PS1080) to obtain depth information. In this investigation, six subjects were monitored while lying on a bed. The value obtained for respiratory rate from the depth camera was compared to that from visual counting. The volunteers breathed for periods of 1 and 3 min, and at rates ranging from 14 to 25 breaths/min. Although no rigorous statistical analysis was performed in this limited study, the differences found between the depth camera and the reference RR were all within 1 breath/min. In a single subject study, Procházka et al. [21] investigated the measurement of respiration rate using depth, RGB camera and IR cameras. A limited number of tests were conducted over deep and shallow breathing and at different respiratory rates. The depth RR was compared to RRs from RGB and IR images and all found to be within a 0.26% error. (Note that, for reasons not stated, the authors did not compare the depth RR to a reference—they only compared the IR RR to the Garmin RR.) A real time respiratory monitoring study of 41 clips from nine subjects by Lin et al. [22] utilised a depth camera with the capability of automatically locating the individual and corresponding region of interest (ROI) and then calculating RR. Good results were achieved with a correlation coefficient for RR of 0.98 and RMSE of 0.82 breaths/min when compared to a reference (which is not specified by the authors). It is interesting to note that the technology described by the authors also had the ability to detect multiple ROIs, from a single person or more than one person at a time. Mateu-Mateus et al. [23] studied 20 healthy subjects in a car driving simulator. The RR was referenced against a Respiband Biosignals PLUX thorax plethysmography system. They obtained a global sensitivity of 77.21% and global PPV (positive predictive value) of 80.69%. (Note that this group did not measure RR per se, rather the frequencies associated with individual breaths found within the RV signal which is more variable in nature than longer term averages.) In a small animal study, Rezaei et al. [24] investigated the respiratory rate of restrained rodents when subjected to fear-inducing predatory odours. They found they could measure respiratory rate with an accuracy of 94.8% using a reference RR from visual observation. A preliminary part of the study involved testing a human over different distances where 0.9 m was found to achieve the highest accuracy (=100%) for RR. Although, not a human study per se, it illustrates the small scales at which such technologies can be utilised.

Taken as a whole, these benchtop studies highlight the potential for respiratory rate monitoring using depth sensing equipment.

### 3.2. Respiratory Rate Clinical Healthy Volunteer and Patient Studies

These studies typically include larger scale, more rigorous examinations of the technology within a clinical setting: often they involve IRB approval and comprise a large number of subjects.

Two related experiments were conducted by Yu et al. [25,26] to determine the accuracy of monitoring respiratory rate during sleep using a depth sensing camera. The effects of motion and sleep position were also considered. The first experiment involved eight healthy participants and consisted of each subject changing sleeping position every 15 breathing cycles in order to determine how body position affects the accuracy of a depth camera in calculating RR. The average accuracy for sheet/no sheet and supine/side position scenarios was found to be 86.3% when compared to reference RIP (respiratory inductance plethysmography) bands (where accuracy was defined as the ratio of the total breathing cycles detected by the depth system and those detected by the RIP bands). In the second part of the work, a single participant was monitored in a series of overnight sleep studies, resulting in a total of 42 h of sleep data over a 10-day period. (Five days using a light blanket and 5 days without a blanket.) An average RR accuracy of 92% was achieved in this sleep study. (Note that the authors also found the average accuracy for detecting the head and torso in the depth video to be between 98.4% and 96.4%, respectively.) Martinez and Stiefelhagen [27] assessed 67 healthy patients in a sleep lab study where 3239 segments of data were collected, each 30 s long. They studied various features such as blanket thickness, various sizes of pillows, presence of books, newspaper and magazines, etc. They achieved an accuracy of 88.7% for RR with reference to a thermistor placed under the nose (where accuracy was specified as being within 1 breath/min from the reference). In addition, they detected a reduction in performance of the system during episodes of apnea. The dynamic region of interest was aligned to the bed and centred on the chest area.

A study involving healthy adult patients was performed by Seppanen et al. [28]. They measured the respiratory function of eight volunteer subjects, including respiratory rate. The subjects were instructed to follow a variety of breathing patterns while being monitored. They selected 2.5 cm wide bands as the ROIs for the depth camera in order to mimic chest/abdomen bands used in sleep studies. They compared their results to a spirometer RR and found very small absolute errors of between 0.26% and 0.30%. Bernacchia et al. [29] assessed 10 healthy young adult subjects and found good agreement between the breath periods derived from a Kinect depth sensing system and a spirometer reference. They achieved a 9.7% RMSD for the breath periods between the two devices. During the tests, which lasted only 40 s per acquisition, the subjects were asked to maintain ‘regular respiratory activity’. (Note that this study considered only individual breath periods and not respiratory rate per se, and less difference would have been obtained if they had taken the mean period as the following group did.) A one-person proof-of-principle study by Centonze et al. [30] used a Kinect to continuously monitor a single patient for 8 h and calculated RR with reference to a polysomnographic record. The average error in frequency was calculated to be 0.87%. This study achieved great accuracy for RR during regular breathing frequency, however, the authors stated that when the breathing behaviour is not regular, a dominant frequency is difficult to identify in the power spectrum.

A 14-person protocolised breath-down lab study by our own group at Medtronic [17] measured continuous RR during an acute hypoxic challenge using a Kinect V2 depth camera. The hypoxic challenge consisted of oxygen saturation reduction steps, down from 100% to 70% which elicited a wide range of respiratory rates. A capnograph was used to provide a reference RR. A bias and RMSD of 0.04 and 0.66 breaths/min, respectively, were found. In addition, there was a high correlation between the depth camera and the reference RR (R = 0.99). Clear respiratory patterns induced by the hypoxic challenge protocol were also exhibited in a number of the signals (as shown in the example of Figure 4b).

A number of research groups have concentrated on younger, nonadult populations. Monitoring the respiratory rates of three preterm infants was the focus of a study by Cenci et al. [31] where each infant was assessed in five 30 s intervals. This group reported the mean breath period, rather than RR, but found excellent agreement with the reference derived from ECG impedance pneumography. An overall correlation coefficient of R = 0.95 was found. A study of two children in the PICU (4 months and 1 year old) by Rehouma et al. [32] demonstrated the ability of depth cameras to accurately calculate RR in this patient population. The patients were ventilated, and the ventilator RR was used as the reference over five 1-min data acquisition periods. RMSDs of 0.77 and 0.68 breaths/min were obtained for the two patients. (Note that the authors also completed a preliminary side study on a mannequin which resulted in an RMSD of 0.53 breaths/min.) In another study concerning younger patients, Al-Naji et al. [33] studied five children, aged between 1 and 5 years old, and found excellent agreement between depth-sensing RR and a piezo-belt reference. Correlation coefficients ranging from 0.97 to 0.99 were found depending on background lighting levels and whether bed sheets were used. Bland–Altman analysis was undertaken with limits of agreement ranging from [−0.91 to +1.0] to [−1.3 to 2.3] breaths/min for scenarios with and without blankets. Apnea events were also included in the protocol.

Taken as a whole body of evidence, the clinical studies involving both healthy volunteers and patients described in this section clearly highlight the potential for respiratory rate monitoring using depth sensing equipment.

## 4. Respiratory Volume Analysis

Many researchers have focused their attention on the characteristics of the respiratory volume signal, often determining its correlation with a refence signal from a spirometer or ventilator. The RV signal is the signal obtained by integrating the depth changes across the patient ROI (usually on the chest as described in Section 2 and Figure 1). However, the measurement of characteristic volumes, e.g., tidal volume (peak-to-trough breath measurement in spontaneous breathing) or forced vital capacity (FVC) from the RV signal and its comparison with a reference is less common. This section deals with both characteristic volumes and the RV signal in turn.

### 4.1. Tidal Volume and Other Characteristic Volumes

A few groups have compared the volume calculated from the depth signal against a known patient volume measurement. These are dealt with here. An early study by Aoki et al. [34] involved four healthy volunteers in a sitting position who were instructed to vary their respiratory flow over 180 s measurement epochs while being monitored by a Kinect depth camera. Correlation coefficients of 0.99 were obtained for all four subjects for tidal volume relative to a flow reference obtained using an expiration gas analyser. Although no statistical measures of error were provided in their paper, the per-subject scatter and corresponding Bland–Altman plots with limits of agreement drawn on are provided. Oh et al. [35] studied 10 healthy adult volunteers, comparing their results against a ventilator reference. They obtained a correlation coefficient and mean tidal volume error of 0.98 and 8.1%, respectively, when they combined both spatial and temporal information within their method. At a much smaller scale, Rehouma et al. [32] investigated the tidal volume of two neonatal patients where they obtained a mean RMSD of 5.9 mL between their depth-based method and the ventilator reference. A study of other pertinent respiratory volumes from the RV signal has been conducted by Soleimani et al. [36] who measured lung volume changes in 40 COPD patients using a Kinect V2 camera. For each patient at least three forced vital capacities (FVCs) and three slow vital capacities (SVCs) were recorded using a depth camera. These values were validated against those obtained using a spirometer. Correlation coefficients of 0.999 were found for both SVC and FVC. The mean/standard deviation of the differences was calculated to be 0.029/0.049 and 0.009/0.039 litres for SVC and FVC, respectively. A more recent paper by Soleimani et al. [37] extends this method further and performs full-body plethysmography for pulmonary function testing using two Kinect depth cameras. In another investigation by the same group, Sharp et al. [38] studied 100 patients from a general respiratory physiology laboratory with a variety of lung issues. They investigated the determination of critical lung capacities using a Kinect-based depth system. They found that their method tracked estimated forced vital capacity (FVC) and vital capacity to within ± <1% but forced expiration volume did not demonstrate acceptable limits of agreement, with 61.9% of readings showing more than 150 mL difference.

### 4.2. Respiratory Volume Signal Analysis

Many other authors have only considered the respiratory volume signal against a reference without calculating tidal volume, or any other characteristic volume from it. For example, Transue et al. [39] investigated the measurement of respiratory volume from a Kinect V2-based depth camera system by employing a Bayesian-based neural network gaining high accuracies when compared to a reference spirometer for this four volunteer proof of principle study. A real time respiratory motion monitoring study, involving 10 healthy volunteers by Wijenayake et al. [40] utilised a depth camera and principal component analysis (PCA). The first 100 depth frames from each subject were used in a PCA respiratory model where temporal and spatial noise of the input data was removed. The RV signal was calculated to have a correlation of 0.97 when compared to a spirometer. A laser line scanner was used as truth for depth measurements. On average there was a 0.53 mm error for depth measurements. In a small study, by Lim et al. [41] with a single volunteer, the respiratory volume signal was measured using a depth camera and validated against a respiratory belt. The correlation coefficient was determined to be 0.74 for non-normalised and 0.96 for normalised signals. A further one-person study investigating the RV signal by Nguyen et al. [42] consisted of an automated system to monitor RV during a 30-min sleep study, by applying depth camera technology. The method applied neural networks to establish a relationship between the RV measured from the depth camera and a spirometer. The mean error in volume measurement between the reference and the depth camera was calculated to be 0.02 litres and the max error 0.05 litres. This study was another example of a proof of principle with limited results and statistical analysis. Kempfle et al. [43] assessed seven healthy volunteers, in a study investigating parameters that influence the ability of the depth camera to measure the RV signal. The parameters included distance from the depth camera, area of the torso being measured by the depth camera and the sampling rate. Data was recorded for two breathing rates (0.17 and 0.25 Hz) for 1 and 2 min, respectively. The experiment was repeated at different distance markers from 1 to 4 m. The optimal region of the torso was found to be the chest, with a larger selected area of the chest performing better than a smaller selection. The respiration signal to noise became ‘debilitating’ as the distance to the person approached 4 m. In addition, they found that, as the sampling rate decreased, there was more time for processing but a negative influence on the signal to noise ratio.

Other studies include those by Harte et al. [44] who studied respiratory volume in groups of healthy and cystic fibrosis patients; Samir et al. [45] who compared respiratory volume measurement using both a Kinect V1 and Kinect V2; Ostadabbas et al. [46] who investigated respiratory volume and airway resistance in a study of the correlation of FEV1 derived from a spirometer and depth sensing system; Ernst et al. [47] who completed a study on respiratory motion tracking with a depth camera; Yang et al. [48] who included sleep patients in a study of sleep event detection and respiratory volume; Shan et al. [49] who also investigated respiratory volume signals in the context of stress classification; Kempfle and Van Laerhoven [50] who propose modelling chest elevation to robustly monitor a user’s respiration, whenever users are sitting or standing or the view is occasionally blocked; some smaller scale proof of principle studies by Yu et al. [51], Prochazka et al. [52], and Aoki et al. [53].

### 4.3. Patterns and Apneas in Respiratory Volume Signals

The manifestation of respiratory patterns in the RV signal was demonstrated earlier in Section 2 and the examples of Figure 4 using signals collected by the authors’ group. There are many examples of patterns emerging in the waveform in the literature. For example, Wang et al. [54] studied the detection of unexpected respiratory patterns in adults for the prognosis, diagnosis, and screening for the patients infected with COVID-19 (the novel coronavirus) based on breathing characteristics. They identified a variety of patterns including Eupnea, Bradypnea, Tachypnea, Biots, Cheynes–Stokes breathing, and Central Apnea using a variety of deep learning approaches. They found that a bidirectional gated recurrent unit with bidirectional and attention mechanisms (BI-AT-GRU) model performed best, attaining a 94.5% classification accuracy. They extended this work to include results from various ROIs and window lengths. More details of their work can be found in Wang et al. [55]. Delimayanti et al. [56] investigated the clustering and classification of breathing patterns using a support vector machine. Three patterns were tested from four volunteers: deep and fast breathing, reading aloud and relaxed breathing. They found that a support vector machine provided the most efficient classifier with the highest accuracy for all subjects of over 99%. Niérat et al. [57] found that structured light patterns (SLPs) enabled the detection of different breathing patterns in COPD patients compared with subjects with no respiratory disease. In other work, the ability of a classifier (trained on data from commercially available depth camera systems) to detect sleep apnea events has been reported by Schätz et al. [58]. They obtained 100% accuracy in identifying apneas when compared to a sleep expert as reference in a data set comprising 57 whole night polysomnographic records. In another study of the detection of respiratory events in sleep patients, Yang et al. [59] combined both depth sensing and audio inputs to a classifier. They found 0.4% error rates for identifying the classes (1) central apnea, (2) obstructive/mixed apnea, (3) hypopnea, and (4) other events, using a support vector machine.

## 5. Concluding Remarks

An extensive review of the literature was conducted with a specific focus on the determination of respiratory parameters using depth sensing camera methods. The review began by introducing the concepts involved in deriving information using depth sensing cameras, before explaining its use in monitoring respiratory information. This was followed by key sections on respiratory rate and respiratory volume analysis.

Depth-based RR and TV were found to be generally accurate in all studies reviewed. However, note that most TV studies involved an experimental set up with a clear view of the chest region which was orthogonal to the line of site. In practice the patient may be in a range of postures and/or under blankets. In such cases RR will still perform with high absolute accuracy (i.e., absolute RR will correspond well with the true value) whereas TV may only be able to trend accurately with true tidal volume (i.e., high correlation between the depth-sensing TV and a reference but not a 1:1 correspondence). However, TV trending could prove very useful for monitoring respiratory patterns and following reduction in volumes over time associated with respiratory compromise [60]—an area where there is a clear need for improved monitoring according to the study by Willens et al. [61]. Another important aspect of the technology is that clear patterns, including apneic events, may be discerned from the RV signal. (These are, in fact, localised trends in tidal volume over short periods of time.)

Depth sensing respiratory monitoring technologies may provide a useful adjunct to traditional camera systems which are becoming more prevalent for remote patient monitoring prompted, in part, by the COVID-19 pandemic [62]. Respiratory rate is a ubiquitous physiological parameter in clinical practice with wide ranging utilisation across a range of areas of care and disease states, both inside and outside the hospital environment [63]. Tidal volume is constrained in its use to much more specific use cases as it generally required more obtrusive equipment such as face masks or endotracheal tubes to monitor. However, a simple method for determining tidal volume (or even tidal volume trending) and respiratory patterns, via depth sensing technologies may accelerate the use of this parameter in the wider patient population. It is recommended that further rigorous studies with large subject numbers should be conducted. These should be targeted at areas of care where this technology may prove particularly useful, including the general care floor (GCF), where a significant number of patients may suffer from respiratory issues stemming from a variety of aetiologies which may manifest in cyclical respiratory patterns, tachypnea or bradypnea, or reductions in tidal volume including apneas; the recovery room, where monitoring of the deeply sedated patient could be automated; the sleep clinic where cyclical respiratory patterns including hypopnea and apnea are of interest; the neonatal intensive care unit where minimising the attachment of probes to delicate skin is important, as is the accurate monitoring of the number and severity of apneic events might aid in the better provision of therapeutic interventions. The technology may also prove useful for out-of-hospital applications including home sleep monitoring, home respiratory monitoring or early discharge to home monitoring.

## Data Availability

All reviewed papers are available.
